# No Differences of Immune Activation and Microbial Translocation Among HIV-infected Children Receiving Combined Antiretroviral Therapy or Protease Inhibitor Monotherapy

**DOI:** 10.1097/MD.0000000000000521

**Published:** 2015-03-20

**Authors:** Lola Falcon-Neyra, Omar J. Benmarzouk-Hidalgo, Lola Madrid, Antoni Noguera-Julian, Claudia Fortuny, Olaf Neth, Luis López-Cortés

**Affiliations:** From the Unidad de Enfermedades Infecciosas e Inmunopatologias, Hospital Infantil Virgen del Rocio, Instituto de Biomedicina de Sevilla (LF-N, LM, ON); Unidad Clínica de Enfermedades Infecciosas, Microbiología y Medicina Preventiva, Hospital Universitario Virgen del Rocío/Instituto de Biomedicina de Sevilla (IBiS), Sevilla (OJB-H, LL-C); ISGlobal, Barcelona Ctr. Int. Health Res. (CRESIB), Hospital Clínic - Universitat de Barcelona, Barcelona, Spain; and Unitat d’Infectologia, Servei de Pediatria, Hospital Sant Joan de Déu, Universitat de Barcelona, Barcelona, Spain (LM, AN-J, CF).

## Abstract

This is a cross-sectional study of 15 aviremic chronic HIV-infected children revealing no differences in immune activation (IA; HLA-DR^+^CD38^+^ CD4^+^ and CD8^+^ T cells, and sCD14) and microbial translocation (MT; lipopolysaccharides (LPS) and 16S rDNA) among HIV-infected patients under combined antiretroviral treatment (cART; n = 10) or ritonavir-boosted protease inhibitor monotherapy (mtPI/rtv; n = 5). In both cases, IA and MT were lower in healthy control children (n = 32). This observational study suggests that ritonavir boosted protease inhibitor monotherapy (mtPI/rtv) is not associated with an increased state of IA or MT as compared with children receiving cART.

## INTRODUCTION

Chronic HIV infection, even under long-term suppressive combined antiretroviral therapy (cART), is associated with persistent immune activation (IA), the latter contributing to impaired immunological reconstitution,^1^ higher mortality rates,^2^ and AIDS and non-AIDS-defining illnesses, such as cardiovascular disease and neoplasms.^3,4^ However, ritonavir-boosted protease inhibitor monotherapy (mtPI/rtv) is an effective and safe alternative for simplification in selected patients with HIV infection in adults^5^; however, the experience with mtPI/rtv in children is very limited.^6^ To date, inflammation, microbial translocation (MT), and IA in HIV-infected children receiving mtPI/rtv have not been studied. Thus, the aim of the study was to compare inflammation, MT, and IA in HIV-infected children receiving mtPI/rtv with those on cART, thereby analyzing whether mtPI/rtv therapy is or is not associated with increased immunological, bacterial, and virological biomarkers compared with the later.

## METHODOLOGY

This was a cross-sectional study of aviremic chronically HIV-infected children between November 2011 and May 2012 from a tertiary-care pediatric hospital in Spain. Inclusion criteria to switch from cART to mtPI/rtv included viral suppression (HIV-RNA levels < 50 copies/ml) for at least 6 months while on cART, lack of a history of prior virological failure and no genotypic resistance in protease gene, as well as simplification to reduce nucleoside analogues and non-analogues toxicity in children who need long term antiretroviral therapy. Children were classified according to their antiretroviral regimen: cART *vs.* mtPI/rtv.

Additionally, a control group of healthy patients, matched for age, sex, and race, referred for routine minor surgery, was used. The study was approved by the local ethical Committee and conducted according to the Declaration of Helsinki. Before inclusion, informed consent from parents or legal guardians was obtained for all patients.

Plasma HIV viremia was assessed by quantitative polymerase chain reaction (PCR) (COBAS AmpliPrep/COBAS TaqMan HIV-1 Test, version 2.0, Roche Diagnostic System, Branchburg, NJ, USA). Soluble serum inflammation markers including ultrasensitive C-reactive protein (uCRP), d-dimer, and β-2-microglobulin levels were routinely determined by the hospital Department of Biochemistry.

Cellular IA was measured as the simultaneous expression of HLA-DR and CD38 in both CD4^+^ and CD8^+^ T cells by flow cytometry (FACSCalibur flow cytometer and CellQuest software, BD Biosciences, Madrid, Spain). Systemic IA was assessed by plasma sCD14 levels (marker of monocyte activation), using the Human sCD14 Quantikine ELISA kit (R&D Systems, Abingdon, UK), following manufacturer's instructions. MT was assessed through plasma lipopolysaccharides (LPS), measured by the Limulus amebocyte lysate chromogenic endpoint assay (Lonza, Basel, Switzerland), according to the manufacturer's recommendations. Additionally, plasma 16S rDNA levels were measured, determined by quantitative polymerase chain reaction (PCR) from 200 μL of plasma, as previously described.^7^ Chosen biomarkers were adapted from previous publications in this field.^8–11^

Quantitative variables were summarized using medians and interquartile ranges, whereas absolute values and relative frequencies were used for qualitative variables. The populations were studied to know whether they followed or not a normal distribution. Normally distributed variables were compared with the Student *t* test or analysis of variance. For non-normally distributed variables, nonparametric tests (the Mann–Whitney *U* and Kruskal–Wallis *H* tests) were used to compare the medians between the different groups. Correlations were assessed by means of the Pearson or Spearman tests as adequate. The differences were considered statistically significant for *P* values <0.05. The statistical analyses were performed using SPSS software (v. 19.0, Chicago, IL, USA).

## RESULTS

A total of 47 children were enrolled in the study, including 15 HIV-infected children (10 children on cART and 5 children on mtPI/rtv) and 32 healthy controls. Patients on monotherapy were switched to ritonavir-boosted lopinavir/r (mLPV/rtv) in 4 cases and atazanavir (mATZ/rtv) in 1 case at standard dosages. In the rest of HIV-infected patients, cART regimens were as follows: 8 children received 2 nucleoside reverse transcriptase inhibitor (NRTI), plus a ritonavir-boosted protease inhibitor (PI/rtv) in 5 and a non-nucleoside reverse transcriptase inhibitor (NNRTI) in 3 children. One child was treated with a NRTI/NNRTI/r/PI regimen and 1 patient with a NRTI/integrase inhibitor (INI) bi-therapy.

As shown in Table 1, no differences regarding clinical parameters, such as sex, age, or body mass index were observed between healthy controls and HIV-infected children, either on cART or mtPI/rtv. Moreover, no differences were found in the time on ART and on viral suppression between HIV-infected children receiving cART or mtPI/rtv. Children in the monotherapy group were on this simplification strategy for a median of 28 months (interquartile range [IQR]: 18.4–29.9 months) prior to the enrollment on this study. None of the HIV-infected children suffered from HCV/HBV co-infection and CDC stage did not differ within the 2 HIV groups (data not shown).

**TABLE 1:**
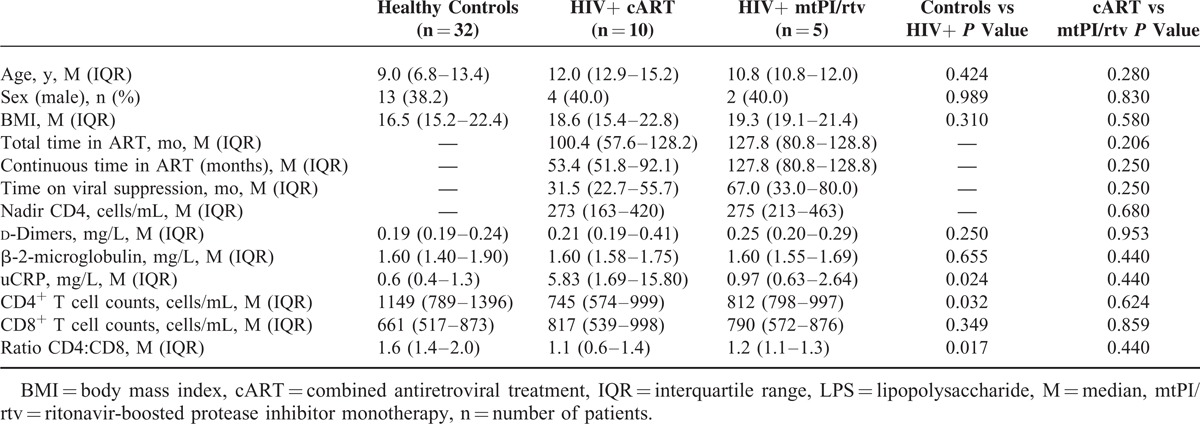
Clinical and Immunovirological Characteristics of the Enrolled Children, Classified as Healthy Controls, HIV-infected Patients on cART and HIV-infected Patients on mtPI/rtv

Regarding inflammation markers, no differences were found between HIV-infected patients on cART and mtPI/rtv in the median (IQR) levels of d-dimers, β-2-microglobulin, or uCRP. In addition, similar results were observed when healthy control and HIV-infected children were compared regarding d-dimer and β-2-microglobulin, though HIV-infected children showed higher uCRP levels (Table 1).

**FIGURE 1:**
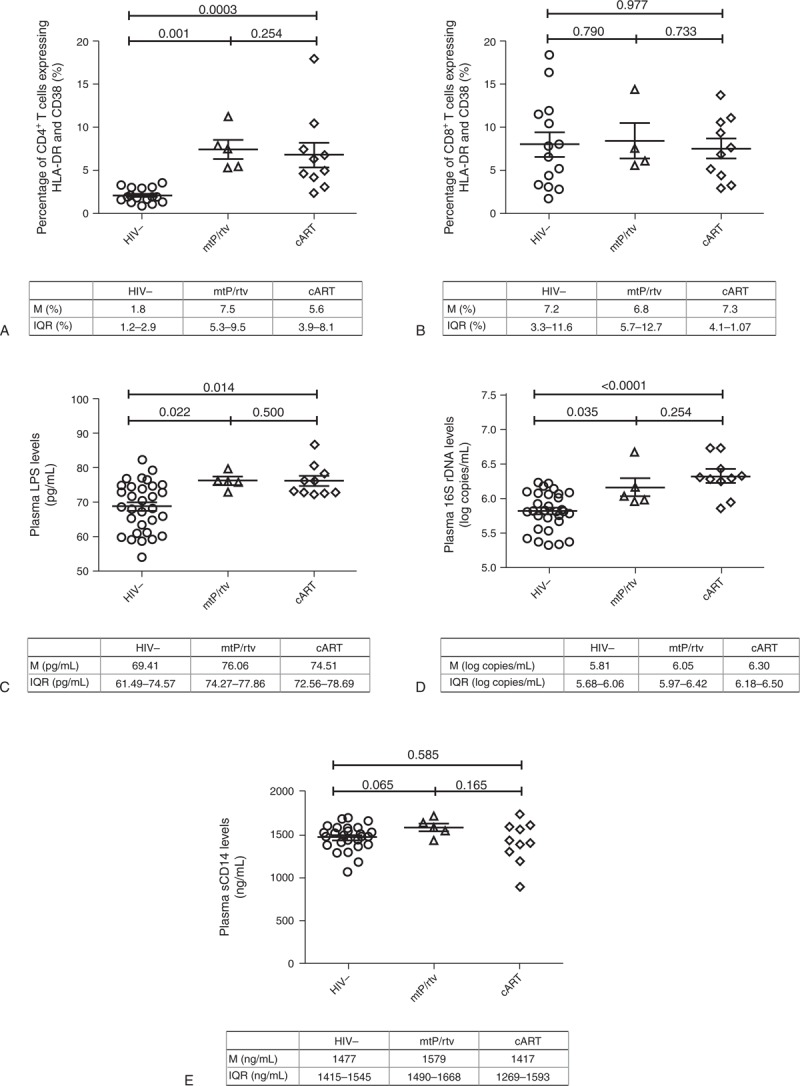
Immune activation (A,B) and microbial translocation (C-E) profiles of HIV negative children (HIV-) and HIV+ children receiving ritonavir boosted protease inhibitor monotherapy (mtPI/rtv) or combined antiretroviral therapy (cART). M = Median, IQR = interquartile range.

As expected, healthy controls showed higher CD4^+^ T-cell counts and CD4:CD8 ratios (Table 1), but lower IA and MT profiles compared with HIV-infected children (Figure 1A–E). Among HIV-infected patients, similar CD4^+^ (745 vs 812 cells/μL, *P* = 0.624) and CD8^+^ T-cells counts (817 vs 790 cells/μL, *P* = 0.859) were found in cART- and mtPI/rtv-treated children, and thus similar CD4:CD8 ratio (1.1 vs 1.2, *P* = 0.440).

Similarly, no differences regarding IA were found in both CD4^+^ (6.3% vs 7.5%,  = 0.221) and CD8^+^ T cells (7.3% vs 6.8%, *P* = 0.733) among children on cART or mtPI/rtv (Figure 1A and B). MT was similar between children on cART and mtPI/rtv assessed by the levels of 16S rDNA (6.3 vs 6.0 log copies/mL, *P* = 0.221) and LPS (74.5 vs 76.1 pg/mL, *P* = 0.460; Figure 1C and D). Finally, plasma sCD14 levels were also similar between both groups of HIV-infected patients (1417 vs 1579 ng/mL, *P* = 0.165; Figure 1E).

## DISCUSSION

Although few studies have focused on inflammation, MT, and IA profiles in HIV-infected children and its potential role on non-AIDS-defining illnesses,^12,13^ no data exist in children receiving mtPI/rtv. Thus, we designed this study, in which higher levels of biomarkers of IA and MT in chronic HIV-infected children compared with healthy controls were observed as previously reported.^12,13^ However, importantly, we did not find differences in inflammation, IA, or MT between aviremic HIV-infected children receiving either cART or mtPI/rtv.

Our observation is supported by recently published data from adult patients receiving mtPI/rtv or cART, demonstrating no differences in the levels of inflammatory and procoagulant markers including CRP, interleukin-6, fibrinogen and d-dimer.^14^ Furthermore, in a 2-year follow-up study of adult patients who were switched from cART to mtPI/rtv, cellular IA (measured as the expression of HLA-DR^+^CD38^+^ on CD4^+^ and CD8^+^ T cells) and systemic IA (measured as soluble CD14 and d-dimer) markers did not change among patients remaining on viral suppression along follow-up.^8^ Owing to a higher risk of viral rebound, the International Antiviral Society-USA Panel does not recommend the use of mtPI/rtv in adult patients when other options are available.^15^ Although preliminary results of the PIVOT (Protease Inhibitor monotherapy Versus Ongoing Triple-therapy in the long-term management of HIV infection) Trial study revealed a viral load rebound rate of 35% using mtPI/rtv,^16^ the clinical relevance of viral load rebounds remains to be established as “blip episodes” and intermittent viremia have been shown not to affect the cellular HIV reservoir dynamic in patients receiving mtPI/rtv and were not associated with accumulation of new resistant mutations.^5,17^

An important limitation of this observational study is the small sample size. However, to date, there are few data on children receiving mtPI/rtv therapy and no published data on immune activation and/or inflammation in this particular group of patients. In this context, our observation is new and probably reassuring, although the presented results need to be confirmed with larger prospective clinical studies. As cART has been and continues to be the gold standard in the management of HIV-infected children, mtPI/rtv therapy is generally not recommended in children and should only be considered in very selected children under exceptional circumstances. It is for these reasons that sample size is and will continue to be low. Therefore, a multicenter study approach is required to further evaluate our observation of mtPI/rtv not to be associated with increased IA or inflammation compared with cART. The clinical relevance of our observation is potentially important, since safety and effectiveness of simplification strategies based on mtPI/rtv as an alternative in very selected children are paramount.
